# A Modified FLT3 PCR Assay Using a TapeStation Readout

**DOI:** 10.3390/genes16060684

**Published:** 2025-05-31

**Authors:** Elizabeth Adele Blake, Madhurya Ramineni, Zoltán N. Oltvai

**Affiliations:** Department of Pathology and Laboratory Medicine, University of Rochester Medical Center, Rochester, NY 14627, USA; elizabetha_blake@urmc.rochester.edu (E.A.B.); madhurya_ramineni@urmc.rochester.edu (M.R.)

**Keywords:** TapeStation, FLT3-ITD, FLT3-TKD, post-PCR fragment analysis, automated gel electrophoresis, ScreenTape assay, electropherogram, very short inserts, large ITD, capillary electrophoresis

## Abstract

**Background:** FLT3 mutation testing is a key ancillary molecular assay for diagnosing and managing patients with acute myeloid leukemia (AML), including assessing the utility of FLT3 inhibitors during induction chemotherapy. FLT3 PCR utilizing fluorescently labeled primers and capillary electrophoresis readout is the most used technique for the rapid detection of FLT3 internal tandem duplications (ITDs) (including very small ITDs) and tyrosine kinase domain (TKD) mutations. However, capillary electrophoresis (CE) is a relatively lengthy and technically demanding result readout mode that could potentially be replaced by faster alternatives. **Methods:** Here, we describe the validation of a modified FLT3 PCR assay that uses the Agilent 4200 TapeStation platform for result readouts. This platform generates quantifiable electropherograms and gel images in under two minutes and at a low cost. We validated its ability to detect FLT3-ITD and -TKD mutations using 22 and 18 previously tested patient samples, respectively. **Results**: The TapeStation 4200 instrument is 100% sensitive, specific, and highly reproducible for post-PCR fragment analysis in detecting FLT3-ITD (greater than 15 bp in size) and TKD mutations in AML patients. Its results are nearly 100% concordant with those obtained from our previously validated NGS and PAGE methods. However, the limitation of this readout mode is its inability to reliably detect FLT3-ITDs smaller than 15 bp in size. **Conclusions**: Given the widespread use of TapeStation instruments in molecular diagnostics laboratories as part of next-generation sequencing (NGS) workflows, this modified assay is well-suited as a companion test for rapid NGS platforms to detect larger FLT3-ITDs, which are NGS often miscalledor under-called by the NGS bioinformatics algorithms. However, it is not suitable for use as a standalone assay, as it is unable to reliably detect very short FLT3-ITDs.

## 1. Introduction

FMS-related tyrosine kinase-3 (FLT3) is a receptor tyrosine kinase expressed on hematopoietic stem cells and early myeloid and lymphoid progenitor cells. Its physiological interaction with the FLT3 ligand activates the PI3K, STAT5, and RAS signaling pathways, which promotes cell survival, proliferation, and differentiation [1]. The FLT3 receptor is encoded by the *FLT3* gene on chromosome 13, which consists of 24 exons and spans approximately 96 kb in length [2,3]. Constitutive activation of the FLT3 receptor is found in up to 30% of newly diagnosed acute myeloid leukemia (AML) patients, primarily due to two types of mutations that enhance downstream signaling and promote leukemogenesis [4,5]. The most common FLT3 mutations are internal tandem duplications (ITDs), which are in-frame insertions of a sequence of nucleotides that occur within the juxtamembrane domain (JMD) of the receptor (in approximately 25% of AML cases). Less frequently, point mutations in FLT3’s second tyrosine kinase domain (TKD) occur in approximately 5–7% of cases [1]. The latter most commonly involves codon 835 (e.g., p.Asp835Tyr), with a few cases reported for the mutation or deletion of codon 836 [6].

The presence of FLT3-ITD, a driver mutation, is associated with a high leukemic burden, significantly increased risk of relapse, and reduced overall survival. Consequently, AML patients with FLT3-ITD mutation(s) often require allogeneic hematopoietic stem cell transplantation in the first remission [7]. These patients and those with FLT3-TKD mutations also benefit from adding FLT3 inhibitors, such as midostaurin, to induction chemotherapy [8,9,10]. Given these clinical implications, the National Comprehensive Cancer Network and European LeukemiaNet guidelines recommend routine molecular testing for *FLT3* mutations in all AML patients as part of the diagnostic workup [11,12] and for consideration in clinical trial enrollment [11].

For providing rapid *FLT3* mutation assay results, polymerase chain reaction (PCR) fragment analysis is the preferred technique for detecting ITD and TKD mutations [7]. The most commonly used PCR assays are based on the design reported by Murphy et al. over 20 years ago [13]. This assay simultaneously tests for the presence of both ITDs and codon 835/836 (i.e., TKD) mutations using PCR amplification with fluorescently labeled primers (and, for TKD mutations, subsequent restriction enzyme digestion) followed by fragment analysis using capillary electrophoresis (CE) readout to determine the size of the post-PCR amplicons [13]. CE provides precise ITD mutation sizing and allows wild-type (wt) -to-mutated peak ratio calculation that may provide prognostic information [13,14,15]. Alternatively, polyacrylamide (slab) gel electrophoresis (PAGE)-based readout can also be used [16], although it does not allow precise ITD sizing or determination of ITD to wt peak ratio.

However, both CE and especially PAGE are relatively lengthy and technically demanding result readout modes. Therefore, to simplify the assay and further reduce its turnaround time, here we present a modification of the standard FLT3 PCR assay. This modification involves using the Agilent TapeStation system for result readout, which outputs electropherograms (EPGs) and gel images in less than two minutes with only 1–2 µL of a sample. As TapeStations are widely used in molecular diagnostics laboratories as part of next-generation sequencing (NGS) protocols, they could also be effectively repurposed for other applications, such as shown here for rapid and cost-effective FLT3 PCR testing.

## 2. Materials and Methods

We validated a modified PCR assay using the TapeStation instrument (Agilent Technologies, Santa Clara, CA, USA) to detect FLT3-ITD and -TKD mutations using 22 and 18 previously tested patient samples, respectively. All patient specimens used in the validation study previously underwent routine FLT3-ITD and -TKD mutation assays by PCR, followed by a PAGE-based readout [16] and subsequent NGS testing.

The modified PCR assay consists of the following steps for the detection of ITD and TKD mutations: (1) DNA isolation; (2) DNA quantification by spectrophotometry; (3) PCR with two sets of primers, one set for ITD and one set for TKD mutations; (4) *Eco*RV digestion of PCR amplicons, performed only for TKD mutation detection; (5) fragment analysis using the TapeStation instrument; and (6) result interpretation by the TapeStation analysis software (Agilent TapeStation Controller Software, version 5.1).

Steps 1 and 2 included DNA isolation and quantification: Peripheral blood and bone marrow samples of at least 0.5 mL received in EDTA vacutainers (blue top) at 2–8 °C were used for the assay. Frozen or severely hemolyzed samples received without appropriate patient identifiers and incomplete or no requisition forms were rejected. DNA extraction was performed using the Qiagen QIAamp DNA Mini Kit (Qiagen, Hilden, Germany) or a Promega Maxwell^®^ RSC Extractor using the Blood DNA Kit (Promega Corporation, Madison, WI, USA). The DNA concentration was measured post-extraction using the NanoDrop One Spectrophotometer (Thermo Fisher Scientific, Wilmington, DE, USA).

Step 3 included the PCR setup: ITD variants predominantly involve exon 14 of the *FLT3* gene and sometimes a portion of exon 15. The first set of primers detects ITDs of varying sizes, ranging from 15–204 bp, including those extending into exon 15 [17,18,19]. The primers used were FLT3-11F (forward primer) and FLT3-11R (reverse primer) (Table 1), aligning with the previous identification of exon 14 as exon 11 [18]. The second primer set was designated as FLT3-AF and FLT3-AD (Table 1). Two master mixes were prepared for the FLT3-ITD and -TKD mutation assays with an optimum DNA input of 50 ng. The DNAs were amplified using different thermal cyclers: MasterCycler Model 53331 (Eppendorf, Hamburg, Germany), VeritiPro Thermal Cycler and ABI 9700 (Applied Biosystems, Foster City, CA, USA), and PTC-100 Thermal Cycler (MJ Research, Waltham, MA, USA).

Step 4 included *Eco*RV digestion: The wild-type sequence at codon 835 of the *FLT3* gene is at an *Eco*RV recognition site (GATATC). Missense mutations in codons 835 or 836 alter the *Eco*RV recognition site, and this biological consequence of the mutation itself is exploited for the assay. Therefore, post-PCR, the *Eco*RV restriction enzyme was added to the TKD test’s PCR product, and the tubes were incubated at 37 °C for at least 12–24 h to ensure complete restriction site digestion. The PCR products for ITD detection were also incubated at room temperature for at least 2 h before being directly loaded onto the TapeStation for fragment analysis.

Step 5 included TapeStation automated electrophoresis: The TapeStation 4200 System (Agilent Technologies, Santa Clara, CA, USA) is an automated electrophoresis instrument that uses the Agilent D1000 ScreenTape assay (Agilent Technologies, Santa Clara, CA, USA) to analyze DNA fragments from 35 to 1000 bp in size. The samples were loaded onto the ScreenTape devices and inserted into the TapeStation 4200 deck. A D1000 buffer containing the upper and lower DNA markers was loaded into wells in the TapeStation instrument. The analysis software outputs EPGs and gel images. The amplicon sizes of the test samples were compared against the electronic ladder and upper and lower DNA markers (Supplemental Figure S1). The software automatically assigns base pair (bp) lengths to each peak and some quantitative data regarding the concentration, molarity, and percentage of the incorporated area. The results obtained from TapeStation were validated by comparing them with those of prior PAGE and/or NGS using the TruSight Myeloid Sequencing Panel on the MiSeqDx platform (Illumina, San Diego, CA, USA) (Illumina TruSight Myeloid Sequencing Panel cat #FC-130-1010) or the Oncomine Myeloid Assay on the Genexus platform (ThermoFisher, Wilmington, DE, USA).

Step 6 included interpretation: *Controls*: Every run included positive- (i.e., sensitivity), negative-, and no-template controls (NTC). The positive controls for ITD and D835 mutations were prepared at 4% and 5%, respectively. A run was deemed acceptable only if the NTC lacked any signal.

*ITD mutations*: The germline band size using the 11F/11R primers is approximately 150 bp in size (Supplemental Figure S2A and lane H3 in S2B). The germline peak size was determined by testing 42 patients and control samples with an acceptable range of 144–156 bp (±3 standard deviations). The size and shape of the germline peaks (whether broader or narrower) can be influenced by the variability in PCR amplification and the amount of sample pipetted into the TapeStation. Any additional larger peak(s) indicate an ITD (Supplemental Figure S2B, including lane A4).

*Codon 835/836 mutations*: The size of the undigested wild-type amplicon is approximately 184 bp (Supplemental Figure S3A and lane F4 in S3D). Amplicons generated from wild-type DNA post-*Eco*RV digestion are approximately 94, 56, and 25 bp in size (Supplemental Figure S3B and lane F6 in S3D). However, the 25 bp band coincides with the low-molecular-weight marker and is thus not assessed in the analysis. TKD mutations at codons 835 or 836 create restriction fragments of approximately 54, 92, and 156 bp in size (Supplemental Figure S3C and lane G6 in S3D) post-*Eco*RV digestion (while the undigested sample, just as the wt, shows a single peak at approximately 184 bp (see lane G4 in Supplemental Figure S3D). The absence of this peak post-digestion indicates the completeness of the restriction site digestion. A positive result is acceptable only if the 156 bp band has a relative intensity more than or equal to the respective band of the 5% sensitivity control.

## 3. Results

### 3.1. Outline of the Validation Strategy

We evaluated the TapeStation’s performance in detecting FLT3-ITDs of varying sizes, including those with low variant-allele frequency (VAF) on NGS. We also tested its ability to detect different point mutations at codons 835/836, including those with low VAF. We assessed the concordance with standard PAGE readout-based PCR and NGS results (Supplemental Table S1 and Table S2), as well as sensitivity, specificity, and intra-run and inter-run reproducibility (Supplemental Table S3 and Table S4) by testing positive and negative samples in triplicates.

For the FLT3-ITD assay, to assess the intra-run reproducibility, three positive and three negative patient samples, along with one each of positive and negative controls and NTC, were amplified in triplicates in the same PCR run. For the inter-run study, five patient samples, one each of positive and negative controls, and NTC were amplified in multiple (2–3) different PCR runs on different (2–3) days.

For the FLT3-TKD mutation assay, six patient samples, one positive and negative control, and NTC were amplified in the same PCR run and digested by *Eco*RV overnight to assess the intra-run reproducibility. All the undigested products and the digested products of the controls were analyzed in a single replicate, and the digested products of the patient samples were analyzed using the TapeStation. For the inter-run study, five patient samples, one each of positive and negative controls, and NTC were amplified in two different PCR runs and digested by *Eco*RV on different days.

### 3.2. Validation of the FLT3-ITD Assay with TapeStation Readout

We tested a total of 22 patient DNA samples for ITD mutations using the FLT3 PCR with either TapeStation readout, PAGE readout, or, at times, by NGS. The runs included positive sensitivity controls (4% MV4-11-cell-line-derived DNA that harbors a FLT3-ITD mutation (ATCC, Manassas, VA, USA), NTC, and negative controls (prepared from previously tested pooled *FLT3*-mutation negative samples). TapeStation analysis identified 10 FLT3-ITD positive, 3 weak positive, and 9 negative cases, showing 100% concordance with the corresponding PAGE and/or NGS results (Supplemental Table S1). Thus, the sensitivity and specificity of the TapeStation for the FLT3-ITD mutation detection are 100%. Supplemental Table S3 summarizes the intra-run and inter-run reproducibility of the FLT3-ITD assay, showing 100% concordance between replicates and multiple runs on different days by different technologists.

**Figure 1:**
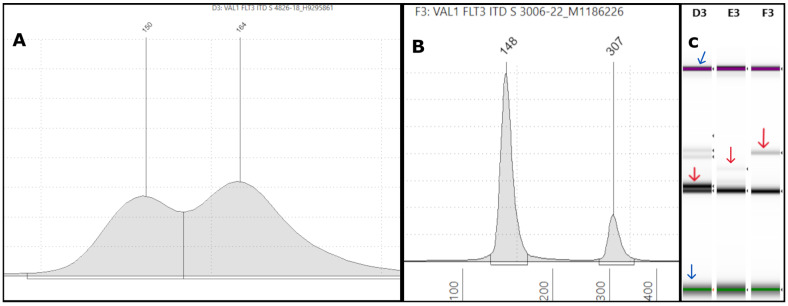
**Representative FLT3-ITD TapeStation results:** (**A**) an electropherogram (EPG) of sample #4826-18 with a 164 bp peak forming a shoulder near the 150 bp germline peak, indicative of a smaller ITD, is shown. The intensity of the peak is high, suggesting a high variant allele frequency (VAF). The corresponding NGS showed a 15 bp ITD with a 40% VAF. (**B**) On the left side, the EPG of sample #3006-22 shows a high-molecular-weight peak at 307 bp. Corresponding NGS revealed a large 153 bp ITD in exon 15 with a 6% VAF. (**C**) The corresponding gel image shows in lane D3 (sample #4826-18) a 164 bp band (red arrow) with an intensity close to the 150 bp germline band, correlating with the high VAF of 40% on NGS. Lane F3 (sample #3006-22) shows a faint band well above the germline band (red arrow), consistent with the large 307 bp ITD of a low VAF (6%) by NGS. Lane E3 shows another test sample with a 227 bp ITD seen as a faint band (red arrow) above the 150 bp germline band. The lowermost and the uppermost bands represent the lower and upper DNA markers, respectively (blue arrows).

Among the FLT3-ITD positive samples, based on the NGS analysis, the length of ITDs ranged from a minimum of 15 bp to a maximum of 204 bp. All of them were identified by the TapeStation, indicating its ability to detect ITDs of varying sizes (see Supplemental Table S1). For example, Figure 1A depicts an ITD with a high VAF (sample #4826-18) but one that is smaller in size. Separately performed NGS on the same sample showed an ITD of 15 bp in length with a VAF of 40% (Supplemental Table S1). In another specimen (sample #3006-22), TapeStation detected an ITD with a peak at approximately 307 bp (Figure 1B). By NGS, we found a large ITD in exon 15 of the *FLT3* gene with 153 bp in length and a 6% VAF (Supplemental Table S1). The corresponding gel pictures for these results are shown in Figure 1C.

**Figure 2:**
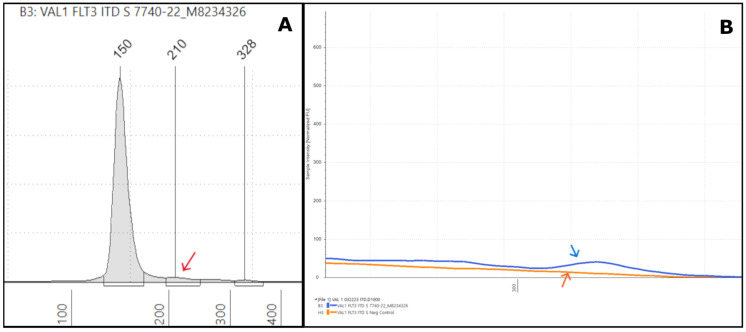
**Representative low-VAF FLT3-ITD TapeStation results:** (**A**) an electropherogram of sample #7740-22 shows a weak positive ITD with a low-intensity peak at 210 bp (red arrow), which was 63 bp in size with a <0.5% VAF by NGS. (**B**) The overlay image shows a similar but slightly higher intensity for the ITD (blue arrow) than the negative control (orange arrow).

The stated sensitivity of the FLT3-ITD assay is 4%, which means that ITDs with as low as a 4% VAF on NGS can be confidently detected by the TapeStation. Out of the 22 cases, four samples had a VAF of <4% by NGS (Supplemental Table S1). All these FLT3-ITDs were identified by the TapeStation, thus demonstrating that it is a reliable tool for detecting low-level or weak positive ITDs (Supplemental Figure S4A–D). The low-level ITDs detected by TapeStation were also positive by PAGE, thus demonstrating a 100% concordance for weak positives or low-level ITDs with both PAGE and NGS assays. For example, TapeStation identified a very low-intensity peak at 210 bp (Figure 2A) for sample #7740-22, which was also reported as a weak positive result by PAGE. The intensity of the peak was only slightly higher than that of the negative control, as seen in the overlay image (Figure 2B). NGS revealed a 63 bp ITD with less than a 0.5% VAF (Supplemental Table S1).

**Figure 3:**
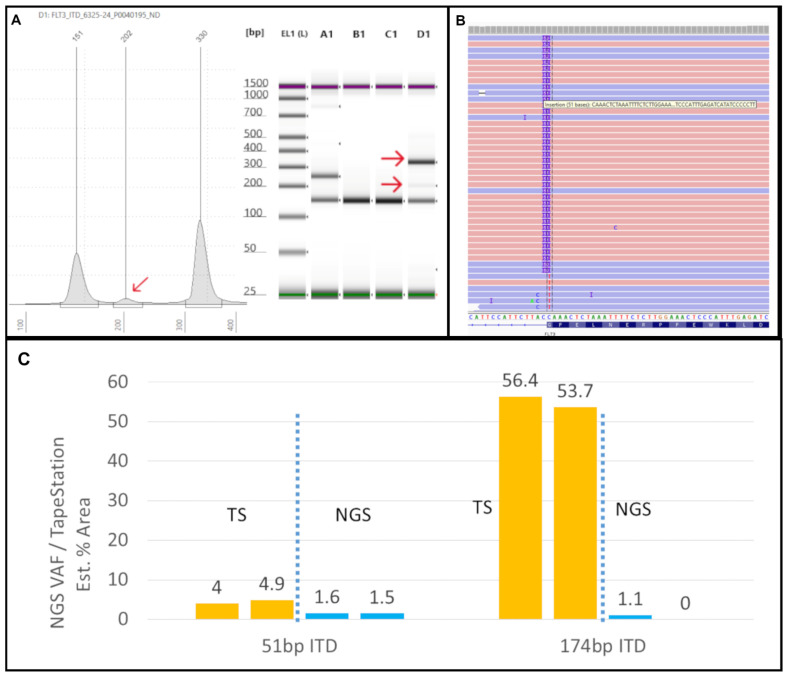
**TapeStation uncovers low-VAF FLT3-ITD not detected by NGS:** (**A**) On the left, an electropherogram (EPG) of sample #6325-24 shows two ITDs of 202 (red arrow) and 330 bp size. On the right, the corresponding gel image in lane D1 shows faint and bright bands at 202 and 330 bp, respectively (red arrows). (**B**) The Integrated Genome Viewer (IGV) image for sample #6325-24 shows only the 51 bp ITD at a VAF of 1.5%. (**C**) The two ITD peaks are clear in the TapeStation (TS) EPG (run in duplicate). By NGS, the 51 bp ITD was present at 51 bp in IGV, but the 174 bp ITD was not found at all in IGV (0%) and was only detected by the Genexus OMAv2 software (1%).

We also noted significant discrepancies in the apparent VAFs when comparing the Tapestation and NGS-based readouts. Specifically, we observed a VAF drop-off that can result in false-negative NGS ITD results with increasing ITD size, especially for ITDs over ~100 bp in length. Misalignments can commonly occur depending on the bioinformatics process in place for NGS data analysis. This can cause either real ITDs being missed or NGS calling multiple ITD mutations when only one is present. For example, sample #6325-24 had two ITD mutations, one at 51 bp, and another one at 174 bp (Figure 3A). The 51 bp ITD was called at a VAF of 1.6% by NGS (Figure 3B) and at approximately 4–5% by TapeStation (Figure 3A), which were largely concordant (in Figure 3C, note that the TapeStation for clinical specimens is run in duplicate). However, the 174 bp ITD was erroneously called twice by NGS due to a misalignment, leading to two ITDs being called at 173 bp and 174 bp. These were called at VAFs of 0.6% and 0.5% (a combined VAF of 1.1%), both values being below the 1% cut-off we routinely use for calling indels ≥3 bp. However, based on the % area calculated from the TapeStation result (Figure 3A), the true VAF appears to be much closer to 50% for the 174 bp ITD (Figure 3C). Indeed, we observed increasing VAF drop-offs during validation of this NGS assay (Genexus Oncomine Myeloid Assay v2, ThermoFisher) for ITDs larger than approximately 70 bp [20]. Thus, using the TapeStation readout-based PCR assay ensures that large ITDs are not missed and that the true number of ITD mutations is counted.

**Figure 4:**
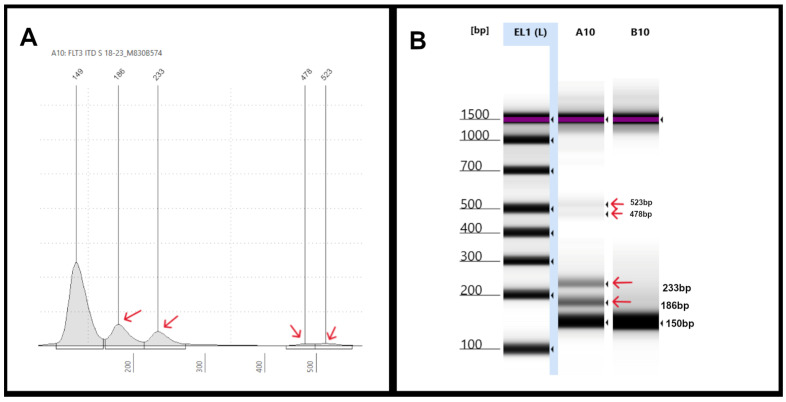
**Representative TapeStation results of samples with multiple FLT3-ITDs:** (**A**) an electropherogram (EPG) of sample #18-23 shows two ITDs with peaks at 186 bp and 233 bp (red arrows). Small additional peaks were also seen at 478 bp and 523 bp (red arrows). (**B**) Gel image of sample #18-23 in lane A10 shows two bright bands corresponding to the 186 bp and 233 bp (red arrows) and two faint bands indicative of the non-specific peaks seen on EPG.

Finally, some apparent artifacts can occasionally be observed, similarly to those we have seen in routine clinical testing with a PAGE readout. For example, Figure 4 depicts an EPG of sample #18-23 which showed two ITDs with peaks at 186 bp and 233 bp (red arrows). Small additional peaks were also seen at 478 bp and 523 bp (red arrows) (Figure 4A), which were also evident on the corresponding gel images (Figure 4B). In contrast, NGS only showed two FLT3-ITDs of 36 bp and 84 bp in length, with a combined VAF of 10%. These additional high-molecular-weight peaks could represent non-specific “ghost bands” due to PCR amplification, technical, reagent, and/or sample-related issues.

### 3.3. Validation of the FLT3-TKD Assay with TapeStation Readout

**Figure 5:**
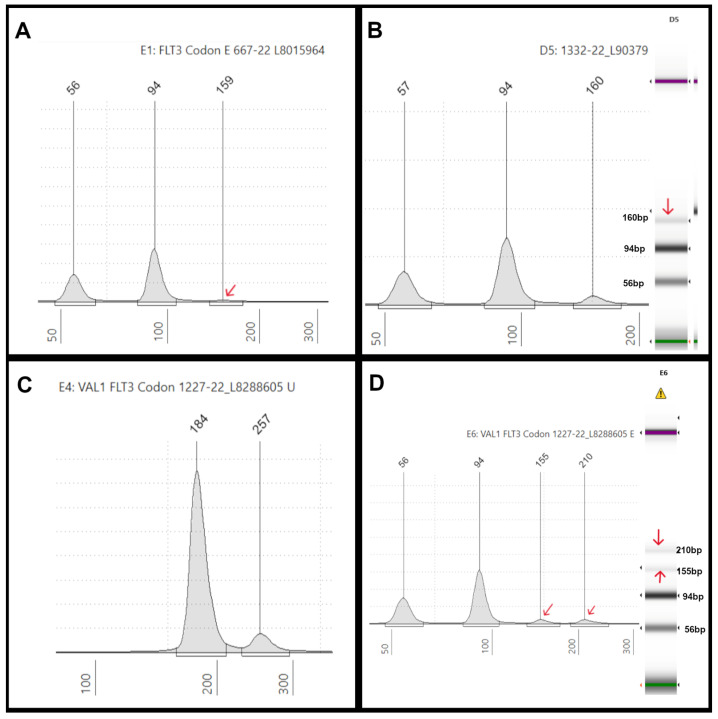
**TKD mutation illustrations:** (**A**) an electropherogram (EPG) of *Eco*RV-digested sample #667-22 displaying three peaks at 56 bp and 94 bp (germline peaks) and 159 bp (mutant peak indicated by the red arrow), which showed a *FLT3*-D835E mutation with a 2% VAF by NGS. (**B**) An EPG of *Eco*RV-digested sample #1332-22 showing three peaks at 57 bp and 94 bp (germline peaks) and 160 bp (mutant peak), which showed a *FLT3*-D835H mutation with a 12% VAF by NGS. On the right, the corresponding gel image in lane D5 shows three bands at 57 bp, 94 bp, and 160 bp (red arrow). (**C**) An EPG of undigested sample #1227-22 shows an extraneous high-molecular-weight peak at 257 bp in addition to the 184 bp peak. (**D**) Sample #1227-22 after *Eco*RV digestion shows peaks at 56 bp, 94 bp, and 155 bp (red arrows) as expected, which is similar to the positive control, but also has an extraneous peak at 210 bp (red arrow), which is also seen in the corresponding gel image on the right (lane E6) (red arrows).

We tested eighteen patient DNA samples for codon 835/836 mutations using the TapeStation and/or PAGE methods or, in some cases, by NGS. Twelve samples were negative for a FLT3-TKD mutation, and six were positive for it, with different mutations at codons 835/836 as determined by NGS (Supplemental Table S2). Specifically, we found four different FLT3-TKD mutations (p.D835V, p.D835E, p.D835H, and p.I836del) by previous NGS (Supplemental Table S2). The TapeStation method identified all of them, thus demonstrating 100% concordance with NGS results. Moreover, while the assay’s stated sensitivity is 5%, the TapeStation readout could detect one sample with a 2% VAF (Supplemental Table S2) (Figure 5A). Based on the validation study mentioned above, the sensitivity and specificity of the TapeStation codon 835/836 mutation analyses are 100%.

The results of intra-run and inter-run reproducibility of the 835/836 codon mutation assays are summarized in Supplemental Table S4. All the tested samples showed 100% concordance between replicates and between multiple runs, except one case (sample #3871-20), in which replicate 1 showed a weak positive result while replicate 2 showed a positive result. Upon visual inspection, the mutant peak sizes at around ~160 bp varied in intensity between the two replicates. This sample had a lower VAF (2%) than our positive control (5%), as verified by NGS. As this assay is qualitative, the discrepancy in the duplicate results might be resolved by performing follow-on NGS.

Sample #1227-22 showed a *FLT3* p.I836del variant, that is, the deletion of isoleucine in the activation loop (A-loop) of the FLT3-TKD domain. The EPG of the undigested sample showed an extraneous high-molecular-weight peak at 257 bp in addition to the wt 184 bp peak (Figure 5C). The post-*Eco*RV digestion EPG showed peaks at 56, 94, and 155 bp, as expected, which is similar to the positive control, but also had an extraneous peak at 210 bp (Figure 5D and lane E6).

Finally, three samples with both ITD and TKD mutations (samples #1227-22, #2575-20, and #1332-22) were identified. TapeStation results were concordant with NGS and PAGE in all three cases. Sample #2575-20 was not included in the validation cohort and was identified separately. The molecular and clinical details of these cases are summarized in Supplemental Table S5.

### 3.4. Limitations of the Modified FLT3 Assay in Detecting Very Small FLT3-ITDs

**Figure 6:**
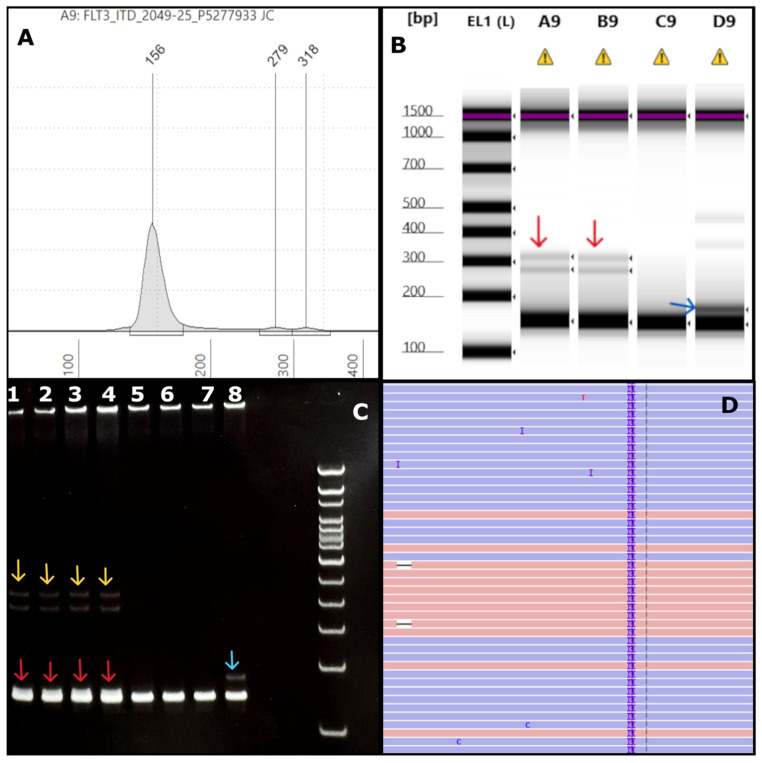
**AML case with very short insertion:** (**A**) an electropherogram (EPG) of sample #2049-25 shows two ITDs of 123 bp and 162 bp in size (279 bp and 318 bp size markers). (**B**) The corresponding gel image in lanes A9 and B9 (sample run in duplicates) shows faint bands close to 300 bp (red arrows) corresponding to the EPG peaks. Lanes D9 and C9 indicate positive (blue arrow) and negative controls, respectively. Note, the image is contrast-enhanced for better visibility of the weak-intensity large ITDs indicated by the red arrows. (**C**) The polyacrylamide gel electrophoresis (PAGE) has the test sample run in duplicates in lanes 1–4, showing faint bands (yellow arrows) and a small insert (red arrows) slightly above the germline band. The other lanes denote positive (lane 8, blue arrow), negative controls (lane 7), and a different test sample (lanes 5–6). (**D**) Integrated Genome Viewer (IGV) image for sample #2049-25 shows only a 6 bp ITD at a VAF of 13%. By NGS, the 123 bp and the 162 bp ITDs were not found at all in IGV (0%) and were not detected by the Genexus OMAv2 software.

Following the validation of the modified FLT3 assay, in our routine clinical practice we identified two cases involving a very short insertion (VSI) and an indel involving the FLT3 juxtamembrane region. This included a bone marrow specimen involved by AML with *RUNX1::RUNX1T1* fusion in 82% of nuclei by FISH. FLT3 PCR using TapeStation readout revealed two faint high-molecular-weight bands, consistent with two large subclonal ITDs (Figure 6A,B). In contrast, the PAGE readout also detected a barely discernible VSI alongside the large subclonal ITDs (Figure 6C). Concurrent NGS analysis identified a likely oncogenic *FLT3* c.1794_1799dup (p.Glu598_Tyr599dup) variant at a VAF of 13% (Figure 6D), as well as oncogenic/likely oncogenic *CBL*, *NRAS*, and two *NF1* variants. A second AML case tested PCR-positive for a FLT3-ITD using both TapeStation and PAGE methods (Supplemental Figure S5A,B), with the latter also uncovering a small *FLT3* deletion (Supplemental Figure S5B). NGS identified a *FLT3* variant of uncertain significance with a 13% VAF (c.1765_1782delinsCCCCTGGTT; p.Tyr589_Phe594delinsProLeuVal) (Supplemental Figure S5C) along with a 94 bp *FLT3-ITD* (VAF: 3%) and oncogenic/likely oncogenic *EZH2*, *KRAS*, *NRAS*, and *PTPN11* variants.

The modified FLT3 assay, which utilizes the Agilent TapeStation platform for result readout, thus has a significant limitation that precludes its use as a standalone assay: its inability to reliably detect FLT3-ITDs 15 bp or smaller (at a 4% detection limit). This limitation stems from the platform’s inherent resolution constraints in analyzing molecular variations of such small sizes. While the assay offers rapid and cost-effective detection of larger ITDs, its sensitivity for smaller mutations remains inferior to traditional methods like CE. Although more technically demanding, CE or PAGE is much better suited to identify very short FLT3-ITDs or complex indels.

## 4. Discussion

Despite the widespread use of NGS in oncology diagnostics, rapid PCR-based testing for FLT3-ITD and -TKD mutations remains a standard practice in many clinical molecular diagnostics laboratories [7,13,21,22]. This is due, in part, to the continued challenge of detecting very large ITDs by NGS [23,24,25] and to quickly informing oncologists of the potential benefit of using FLT3 inhibitors in the induction cocktail for AML patients [7].

Post-PCR fragment analyses are typically performed by CE or less frequently by PAGE. Both methods separate DNA amplicons by charge and size. In PAGE, the DNA molecules move through a slab gel matrix, forming bands that are viewed after staining and compared to a standard. In CE, fluorescently labeled DNA molecules travel through a narrow tube filled with a fluid or gel, and a detector measures the separated components as peaks on an electropherogram for analysis [26].

PAGE provides high separation efficiency at minimal cost, making it one of the most widely used methods, especially in technology-restricted settings. However, the laborious process of PAGE and the increased technician time required are disadvantages for clinical laboratories with high throughput [26]. CE-based methods offer faster separation and higher resolution because the thin tubes have a higher surface-to-volume ratio than slab gels, allowing for quicker heat dissipation and operation at high voltages without overheating. The narrow capillary tubes also enhance separation efficiency by reducing the lateral diffusion of molecules. One of the most significant advantages of CE is its ability to be completely automated, but it comes with a higher cost, and its run time is still non-negligible [27,28,29].

In contrast, the TapeStation performs automated gel electrophoresis and is widely used to assess nucleic acid size, concentration, and integrity throughout the NGS library preparation process. The 4200 TapeStation system can run as many as 96 DNA samples (i.e., one can load six ScreenTape cartridges at a time, with 16 samples per cartridge) on a single plate, which can be completed in less than 20 min while eliminating contamination and carryover. Unlike PAGE, the automated analysis is more reproducible, avoiding the variabilities that could arise from using gels, staining, and UV photography in the PAGE technique. Another significant advantage of the TapeStation instrument is that the ScreenTape cartridges can be stored at 4 °C for up to two weeks and reused any time before the expiry date. Moreover, the TapeStation offers full scalability with a constant cost per sample. A sample quantity as low as 2 µL is sufficient to obtain accurate sizing of the amplicons. In addition, FLT3-ITD detection fits temporally well with rapid NGS protocols (for e.g., the Genexus platform).

In the current study, the TapeStation analysis identified all the ITD mutations detected by NGS and PAGE, demonstrating 100% concordance with these methods. Smaller ITDs are expected to form a shoulder close to the germline peak at ~150 bp because only a smaller sequence of nucleotides is duplicated (Figure 1A and Figure 2A and Supplemental Figures S2B and S4A). As the ITD increases in size, the peak is displaced towards the upper marker (Figure 1B and Supplemental Figure S4B,C). The difference between the ITD and germline peak sizes generally correlates with the ITD size detected by NGS. For example, in Figure 1A, the difference between the germline (150 bp) and ITD peaks (164 bp) is 14 bp, close to the 15 bp size reported by the NGS bioinformatics software. A similar finding could be observed in other cases (Figure 2A and Supplemental Figure S4A,C). However, this observation is only based on individual cases, and we did not perform any statistical correlation between ITD sizes determined by PCR and NGS methods. Spencer et al. identified an excellent concordance between the ITD sizes determined by PCR- and NGS-based methods [21]. However, in one of our samples (#1227-22), TapeStation revealed a 296 bp peak farther from the germline peak, reported as 18 bp on NGS rather than the expected 146 bp (Supplemental Figure S4C). One possibility for this discrepancy is PCR bias, which refers to the preferential amplification of shorter ITD inserts over the longer ones during DNA amplification. This leads to the underrepresentation of larger ITDs in the final DNA library, resulting in inaccurate detection and quantification [30]. Another reason could be due to the misalignment and misassembly of the duplicated sequence by the bioinformatics pipeline, as observed by Spencer et al. [21]. In our laboratory, we use an in-house-developed algorithm for ITD detection by NGS, called “IndelDuper”. It is possible that our in-house-developed pipeline under-called this particular ITD. We identified a recent case (not included in the validation set), #6325-24, which showed ITD misalignment by NGS and VAF drop-off (Figure 3). TapeStation showed two ITDs at 51 bp and 174 bp. The 51 bp ITD was called at 1.6% VAF, which is concordant with the peak height by TapeStation (Figure 3C). However, the 174 bp was called twice by NGS due to a misalignment, leading to ITDs being called at both 173 and 174 bp. These were called at 0.6% and 0.5%, leading to a combined VAF of 1.1%. However, based on the % area calculated from the TapeStation and the prominent peak, the true VAF appears closer to 50%. Indeed, we observed increasing VAF drop-offs for ITDs larger than 70 bp.

The sensitivity of PCR fragment analysis with CE for FLT3-ITD is around 5% [13,14]. In line with the existing literature, the sensitivity of the TapeStation assay is 4%, and it is also effective in detecting weak positive ITDs with peak intensities much lower than the sensitivity control, suggestive of low VAF by NGS (Figure 2A and Supplemental Figure S4A,C). In most cases, low-intensity peaks had low VAFs compared to high-intensity peaks, but we did not perform a statistical analysis to compare the correlation between peak intensity on the TapeStation EPG and VAF by NGS. However, one case (#4902-22) with a large 204 bp ITD showed a high-intensity peak with a VAF of 1% (Supplemental Figure S4B). A more recent sample from the same patient showed the same discrepancy. Since our fragment length for NGS is 300 bp, the ITD could have been significantly under-called by the bioinformatics algorithm due to its inability to align large ITD sequences. Our lowest VAF for ITD, successfully detected by the TapeStation software (Agilent TapeStation Controller Software, version 5.1), is 0.5% (Figure 2). Although we were not confident signing out the very low-intensity cases as frankly positive for ITD, repeat testing and careful manual inspection using the overlay view strengthened our suspicion of an ITD, and we alerted the clinician. In many cases, in correlation with the final NGS result, such cases were confidently signed out as positive. While we reported a 100% concordance with NGS, some studies found a slightly lower concordance using the CE, the most widely used fragment analysis method [21,31]. This could be attributed to the differences in sample sizes, PCR-based methods, and the vast heterogeneity in bioinformatics pipelines used across various molecular labs. In summary, the sensitivity of our TapeStation assay was enhanced by resorting to careful manual inspection of the peaks and using an overlay view for low-level ITDs. The importance of manual review was also reiterated by Spencer et al. [21].

Regarding FLT3-TKD variants, D835Y is the most common codon 835 mutation described in the literature, with a few documented cases of D835H and D835E. Deletion of codon 836 occurred in 10.2% of cases in a large study comprising 3082 patients [32]. TapeStation analysis identified the most common D835Y variant and detected the D835E (Figure 5A), D835H (Figure 5B and lane D5), and I836del variants (Figure 5C,D). The results of TapeStation are 100% concordant with NGS for codon 835/836 mutation analysis.

The I836del variant represents the deletion of isoleucine in the activation loop (A-loop) of the FLT3-TKD domain. Although less common than D835 mutations, it is considered a hotspot due to constitutive activation of the FLT3 receptor [33]. The codon 836 deletion (and another specimen that harbored a *FLT3* D835del) showed a high molecular weight extraneous band on both digested and undigested samples on TapeStation (Figure 5C,D). This is consistent with the results of PAGE and seems unique to these deletion mutations. Although incomplete digestion can cause extraneous peaks, it may not be the case here, as it was also detected in undigested samples. While we do not understand the reason for the presence of the extra peak, it is reproducible and highlights the presence of FLT3 codon 835/836 single-amino-acid deletion mutants.

The TapeStation analysis had an intra-run reproducibility, sensitivity, and specificity of 100% for detecting TKD mutations. For the inter-run reproducibility study, one of the five samples showed a discrepancy with varied peak sizes between the two replicates. Notably, this sample had lower sensitivity (1.6%) than the positive control (5%), as verified by NGS. Such a discrepancy might be resolved by correlating with NGS on low-sensitivity samples. The ability to overlay and compare electropherograms of samples and controls makes visual inspection seamless and allows the detection of weak mutations. In the past, Mehta et al. also developed a home-brew assay for post-PCR fragment analysis using the TapeStation 4200 instrument for detecting MET exon 14 skipping mutations, with a reported concordance of 84.6% with NGS results [34].

Three samples from the overall validation data showed both ITD and TKD mutations, and TapeStation results were concordant with NGS and PAGE results in all three cases (Supplemental Table S5). The prevalence of dual ITD-TKD mutations in AMLs was reported as 1.7% (*n* = 979) and 26% (*n* = 452) in two studies, but their prognostic impact is unclear [6,35]. In an in vitro study, dual FLT3-ITD and -TKD mutations have been implicated in resistance to FLT3 tyrosine kinase inhibitor therapy in AML [36].

A limitation of the modified assay is that very short insertions (VSI) and indels in the FLT3 juxtamembrane domain cannot be detected via TapeStation readout. This limitation is significant, as FLT3 VSIs have been shown to behave similarly to average sized FLT3-ITDs with similar FLT3 inhibitor sensitivity profiles [37]. Therefore, this assay is best suited as a companion test for rapid NGS platforms in diagnosing AML but is not appropriate for standalone FLT3 testing, where CE-based readout remains the standard.

## 5. Conclusions

In summary, the TapeStation 4200 instrument is 100% sensitive, specific, and highly reproducible for post-PCR fragment analysis to detect FLT3-ITD (larger than 15 bp in size) and TKD mutations in AML patients. It is also 100% concordant with our previously validated NGS and PAGE methods. Less manual processing significantly saves technician time and offers faster results (1–2 min per sample). Weak positive ITDs and TKD mutations can be detected by careful manual review of the electropherograms for small peaks and by using overlay electropherograms with further confirmation by NGS. This modified FLT3 PCR assay is well suited as a companion test for rapid NGS platforms to detect larger FLT3-ITDs, which are often miscalled or under-called by the NGS software. However, it is not appropriate for standalone FLT3 testing, where CE-based readout remains the standard.

## Figures and Tables

**Table 1:** Primer sequences used in the assay [13].

Primer Name	Primer Sequence	Position on Sequence in Accession AL445262.7
FLT3-11F	GCAATTTAGGTATGAAAGCCAGC	17,469–17,447
FLT3-11R	CCAAACTCTAAATTTTCTCTTGGAAAC	17,335–17,361
FLT3-AF	GCTTGTCACCCACGGGAAAG	1811–1792
FLT3-AD	AGTGAGTGCAGTTGTTTACCATGATATC	1642–1669

## Data Availability

The consent documentation signed by the patients do not expressly allow submission of full sequencing data (FASTQ, BAM/BAI, VCF) to external data repositories.

## Supplementary Materials

The following supporting information can be downloaded at https://www.mdpi.com/article/10.3390/genes16060684/s1. This study was originally presented at the Association for Molecular Pathology (AMP) Annual Meeting held in Vancouver, Canada, on 20–22 November 2024. Supplementary information, consisting of five figures (Supplemental Figures S1–S5) and five tables (Supplemental Tables S1–S5), are available through the *MDPI* website. Supplementary Table S1: The concordance results for 22 specimens tested by PCR for the presence of ITD mutation(s) are shown. PAGE, polyacrylamide gel electrophoresis; Table S2: The concordance results for 18 specimens tested by PCR for the presence of TKD mutation(s) are shown. PAGE, polyacrylamide gel electrophoresis; Supplementary Table S3: The Intra-run and inter-run reproducibility results for 6 specimens tested by PCR for the presence of ITD mutation(s) are shown, Supplementary Table S4: The Intra-run and inter-run reproducibility results for 6 specimens tested by PCR for the presence of TKD mutation(s) are shown. Supplemental figures: Figure S1: TapeStation software output of electropherograms. Figure S2: FLT3-ITD controls. Figure S3: FLT3-TKD controls. Figure S4: Low-VAF ITD illustrations. Figure S5: AML case with complex indel leading to a small deletion.

## Abbreviations

The following abbreviations are used in this manuscript:

AML	Acute myeloid leukemia
ITD(s)	Internal tandem duplication(s)
JMD	Juxtamembrane domain
TKD	Tyrosine kinase domain
PCR	Polymerase chain reaction
CE	Capillary electrophoresis
wt	Wild type
PAGE	Polyacrylamide gel electrophoresis
NGS	Next-generation sequencing
bp	Base pairs
NTC	No template control
VAF	Variant allele frequency
EPG	Electropherogram
VSI(s)	Very short insert(s)
