# Supplementary material for: A Modified FLT3 PCR Assay Using a TapeStation Readout
Source: Genes (Basel). 2025 May 31;16(6):684. doi: 10.3390/genes16060684 (PMC12192278; doi:10.3390/genes16060684)
Supplement: Supplementary file 1 [file genes-16-00684-s001.zip › Suppl.figure legends.pdf]

Supplemental figures

Figure S1: TapeStation software output of electropherograms

An electropherogram image of the electronic ladder with lower and upper DNA markers of 25bp and 1500bp, respectively, is shown. Between these DNA markers, there are several DNA fragments of known peak sizes against which the DNA size and concentration of test samples are measured.

Figure S2: FLT3-ITD controls

Electropherograms of **(A)** Wild-type FLT3 PCR product (negative control) with a single peak at 150bp; **(B)** 4% sensitivity (positive control) with a 180bp peak (red arrow) forming a shoulder close to the germline peak of 152bp. The corresponding gel image, in lane A4, shows a faint band at 180bp that is very close (red arrow) to the bright germline band, which is 152bp. Lane “EL2” indicates an electronic ladder with multiple bands of varying sizes, like the electronic ladder in the electropherogram.

Figure S3: FLT3-TKD controls

(A) Undigested wild-type sample with a single 184bp peak; **(B)** *Eco*RV digested wild-type sample showing two peaks at 56 and 94bp. There is no peak at 158bp; **(C)** Positive control after *Eco*RV digestion showing a 156bp peak in addition to the peaks at 54 and 92bp; **(D)** Lanes F4 and G4 in the corresponding gel with a single dark at 184bp represents undigested negative and positive controls respectively. Lane F6 (digested negative control) shows 2 fairly dark bands at 56 and 94bp. Lane G6 (digested positive control) shows a third faint band at 156bp (red arrow) in addition to the 56 and 94bp bands.

Figure S4: Low-VAF ITD illustrations

(A) #7633-22 with a low-intensity peak at 214bp (red arrow), which was 60bp in size and 1% VAF by NGS; **(B)** #4902-22 with a high-intensity peak at 362bp, which was 204bp in size and 1% VAF by NGS; **(C)** #1227-22 with a very low-intensity peak at 296bp (red arrow), which showed 2% VAF and 18bp in size by NGS. The corresponding gel image in lane G3 shows a very faint band at 296bp (red arrow). Faint bands in gel electropherograms can be enhanced by adjusting the contrast in the TapeStation analysis software; **(D)** Overlay image of #1227-22. The zoomed-in view with the sample graph in blue shows a distinctly higher peak (blue arrow) than the negative control in orange (orange arrow), thus confirming the presence of ITD.

Figure S5: AML case with complex indel leading to a small deletion

(A) An electropherogram (EPG) of sample #91-25 shows an ITD of 95 bp in size (244 bp size marker) (red arrow). **(B)** The corresponding gel image in lanes A1 and B1 (run in duplicates) show a faint band at 242bp (red arrows). Lane G1 indicates a positive control (blue arrow). Lanes C1, D1 and E1 represent different test samples in the same run. **(C)** PAGE analysis in lanes 3 and 4 shows a distinct band significantly above (yellow arrows) and slightly below the germline band (red arrows). **(D)** The Integrated Genome Viewer (IGV) image for sample #91-25 shows a *FLT3* indel (*FLT3* c.1765_1782delinsCCCCTGGTT, p.Tyr589_Phe594delinsProLeuVal) at a VAF of 13% and a 94 bp FLT3-ITD at a VAF of 3%.
