# Supplementary material for: A Modified FLT3 PCR Assay Using a TapeStation Readout
Source: Genes (Basel). 2025 May 31;16(6):684. doi: 10.3390/genes16060684 (PMC12192278; doi:10.3390/genes16060684)
Supplement: Supplementary file 1 [file genes-16-00684-s001.zip › Table S1 ITD concordance.pdf]

**Sample ID**	**Tapestation Result**	**PAGE Result**	**NGS Result (VAF)**	**ITD size**
# 1227-22	Weak Positive	Weak Positive	Positive (2%)	18 bp
# 4902-22	Positive	Positive	Positive (1%)	204 bp
# 3006-22	Positive	Positive	Positive (6%)	156 bp (exon 15)
# 4826-18	Positive	N/A	Positive (40%)	15 bp
# 7553-22	Negative	Negative	N/A	N/A
# 7567-22	Positive	Negative	N/A	N/A
# 7570-22	Positive	Positive	N/A	N/A
# 7572-22	Negative	Negative	N/A	N/A
# 7800-22	Negative	Negative	Negative	N/A
# 7812-22	Positive	Positive	Positive (39%)	33 bp
# 7823-22	Negative	Negative	N/A	N/A
# 7376-22	Negative	Negative	N/A	N/A
# 7411-22	Negative	Negative	N/A	N/A
# 7438-22	Negative	Negative	N/A	N/A
# 7388-22	Negative	Negative	Negative	N/A
# 4842-22	Positive	Positive	N/A	N/A
# 6719-22	Positive	Positive	N/A	N/A
# 7010-22	Positive	Positive	N/A	N/A
# 1592-20	Positive	Positive	N/A	N/A
# 7740-22	Weak Positive	Weak Positive	Positive (<0.5%)	63 bp
# 7633-22	Weak Positive	Weak Positive	Positive (1%)	60 bp
# 4381-19	Negative	Negative	N/A	N/A
