# Supplementary material for: A Modified FLT3 PCR Assay Using a TapeStation Readout
Source: Genes (Basel). 2025 May 31;16(6):684. doi: 10.3390/genes16060684 (PMC12192278; doi:10.3390/genes16060684)
Supplement: Supplementary file 1 [file genes-16-00684-s001.zip › Table S2 intra and inter run reproducibility ITD.pdf]

	**Intra-run reproducibility**			**Inter-run reproducibility**	
**Sample ID**	**Replicate 1**	**Replicate 2**	**Replicate 3**	**No. of runs**	**Result**
# 626-21	Positive	Positive	Positive	2	Positive
# 7633-22	Weak Positive	Weak Positive	Weak Positive	3	Weak positive
# 5196-20	Positive	Positive	Positive	N/A	N/A
# 665-22	Negative	Negative	Negative	N/A	N/A
# 667-22	Negative	Negative	Negative	2	Negative
# 3871-20	Negative	Negative	Negative		
Positive (4% sensitivity) control	Positive	Positive	Positive	3	Positive
Negative control	Negative	Negative	Negative	3	Negative
NTC	No Signal	No Signal	No Signal	3	No signal
