# Supplementary material for: A Modified FLT3 PCR Assay Using a TapeStation Readout
Source: Genes (Basel). 2025 May 31;16(6):684. doi: 10.3390/genes16060684 (PMC12192278; doi:10.3390/genes16060684)
Supplement: Supplementary file 1 [file genes-16-00684-s001.zip › Table S3 concordance Codon 835.pdf]

**Sample**	**Tapestation Result**	**PAGE Result**	**NGS Analysis**	
			**Result (VAF)**	**Mutation**
# 4381-19	Negative	Negative	N/A	N/A
# 7376-22	Negative	Negative	N/A	N/A
# 7388-22	Negative	Negative	Negative	N/A
# 7411-22	Negative	Negative	N/A	N/A
# 7438-22	Negative	Negative	Negative	N/A
# 7553-22	Negative	Negative	N/A	N/A
# 7567-22	Negative	Negative	N/A	N/A
# 7570-22	Negative	Negative	N/A	N/A
# 7572-22	Negative	Negative	N/A	N/A
# 7800-22	Negative	Negative	Negative	N/A
# 7812-22	Negative	Positive	Negative	N/A
# 7823-22	Negative	Negative	N/A	N/A
# 2575-20	Positive	N/A	Positive (5%)	D835E
# 667-22	Positive	Positive	Positive (2%)	D835V
# 1332-22	Positive	Positive	Positive (12%)	D835H
# 1227-22	Positive	Weak Positive	Positive (7%)	I836del
# 3871-20	Positive	Negative	Positive (2%)	D835H
# 665-22	Positive	Positive	Positive (5%)	D835E
