# Supplementary material for: A Modified FLT3 PCR Assay Using a TapeStation Readout
Source: Genes (Basel). 2025 May 31;16(6):684. doi: 10.3390/genes16060684 (PMC12192278; doi:10.3390/genes16060684)
Supplement: Supplementary file 1 [file genes-16-00684-s001.zip › Table S4 codon reproducibility.pdf]

	**Intra-run reproducibility (digested samples)**			**Inter-run reproducibility (digested samples)**	
**Sample ID**	**Replicate 1**	**Replicate 2**	**Replicate 3**	**No. of runs**	**Results**
**# 626-21**	Negative	Negative	Negative	2	Negative in both runs
**# 7633-22**	Negative	Negative	Negative	2	Negative in both runs
**# 5196-20**	Negative	Negative	Negative	N/A	N/A
**# 665-22**	Positive	Positive	Positive	2	Positive in both runs
**# 667-22**	Weak Positive	Weak Positive	Weak Positive	2	Weak positive in both runs
**# 3871-20**	Weak Positive	Weak Positive	Weak Positive	2	Weak positive in replicate #1 and positive in replicate #2.
