# Supplementary material for: A Modified FLT3 PCR Assay Using a TapeStation Readout
Source: Genes (Basel). 2025 May 31;16(6):684. doi: 10.3390/genes16060684 (PMC12192278; doi:10.3390/genes16060684)
Supplement: Supplementary file 1 [file genes-16-00684-s001.zip › Table S5.pdf]

Sample ID	ITD mutation by NGS	TKD mutation by NGS	Tapestation result	Follow-up
# 1227-22	18bp @ 2% VAF	I836del	Positive for both ITD and TKD	- Deceased within 6 months of diagnosis.<br>- Received 8 cycles of Azacitidine and Venetoclax.
# 2575-20	Unknown size @ 2% VAF	D835E	Positive for both ITD and TKD	- Deceased within 6 months of undergoing a stem cell transplant due to relapsed/refractory AML.<br>- Received 7+3 (Cytarabine + Daunorubicin) +Midostaurin before transplant.
# 1332-22	69 bp @ 38% VAF	D835H	Positive for both ITD and TKD	- Deceased due to relapsed AML 2 years post-transplant while on maintenance with Gilteritinib and Venetoclax.
