# Supplementary figures and images for: A Modified FLT3 PCR Assay Using a TapeStation Readout
Source: Genes (Basel). 2025 May 31;16(6):684. doi: 10.3390/genes16060684 (PMC12192278; doi:10.3390/genes16060684)

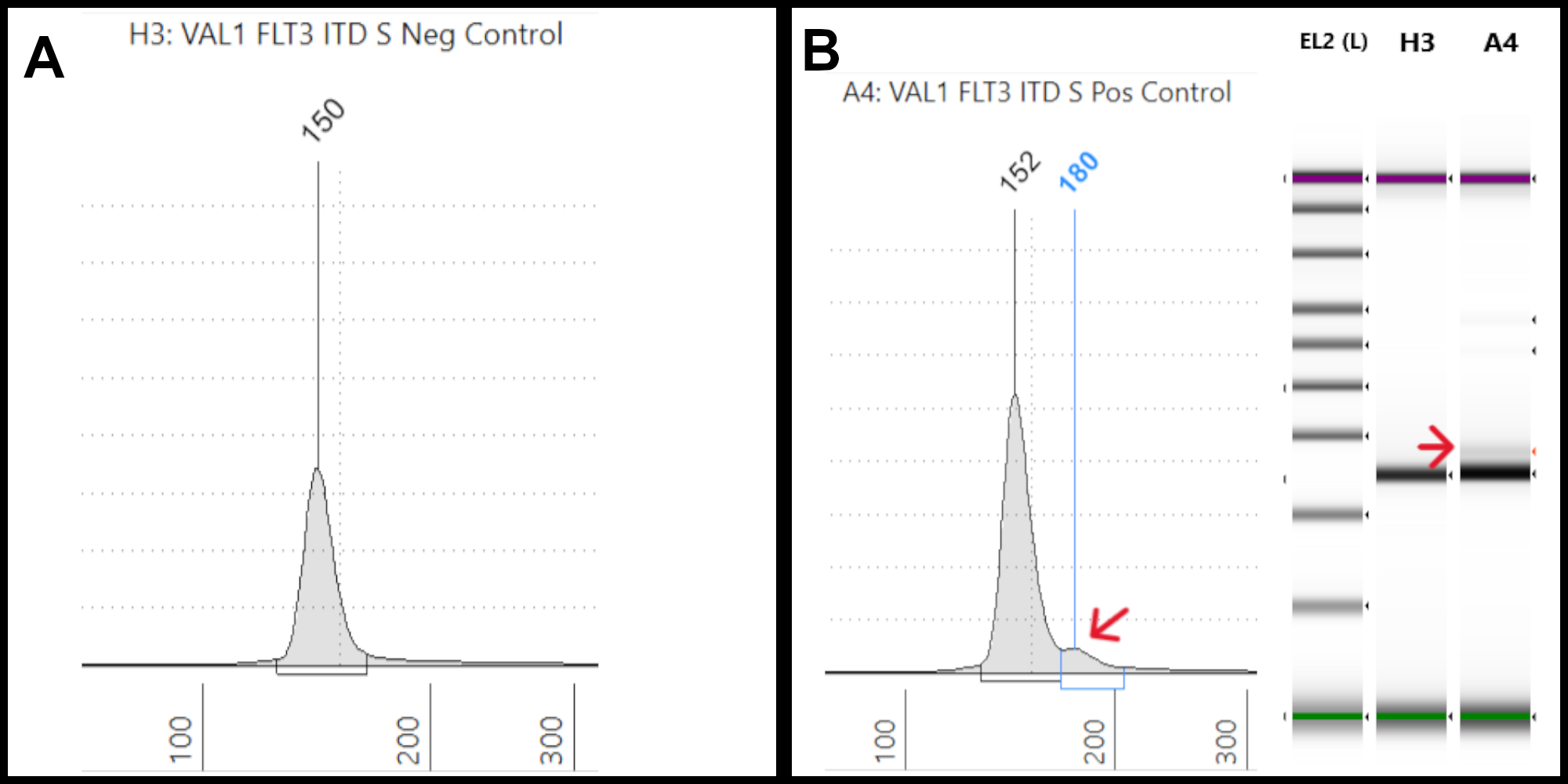

Supplement: Supplementary file 1 [file genes-16-00684-s001.zip › Supp fig 2 ITD controls.tif]

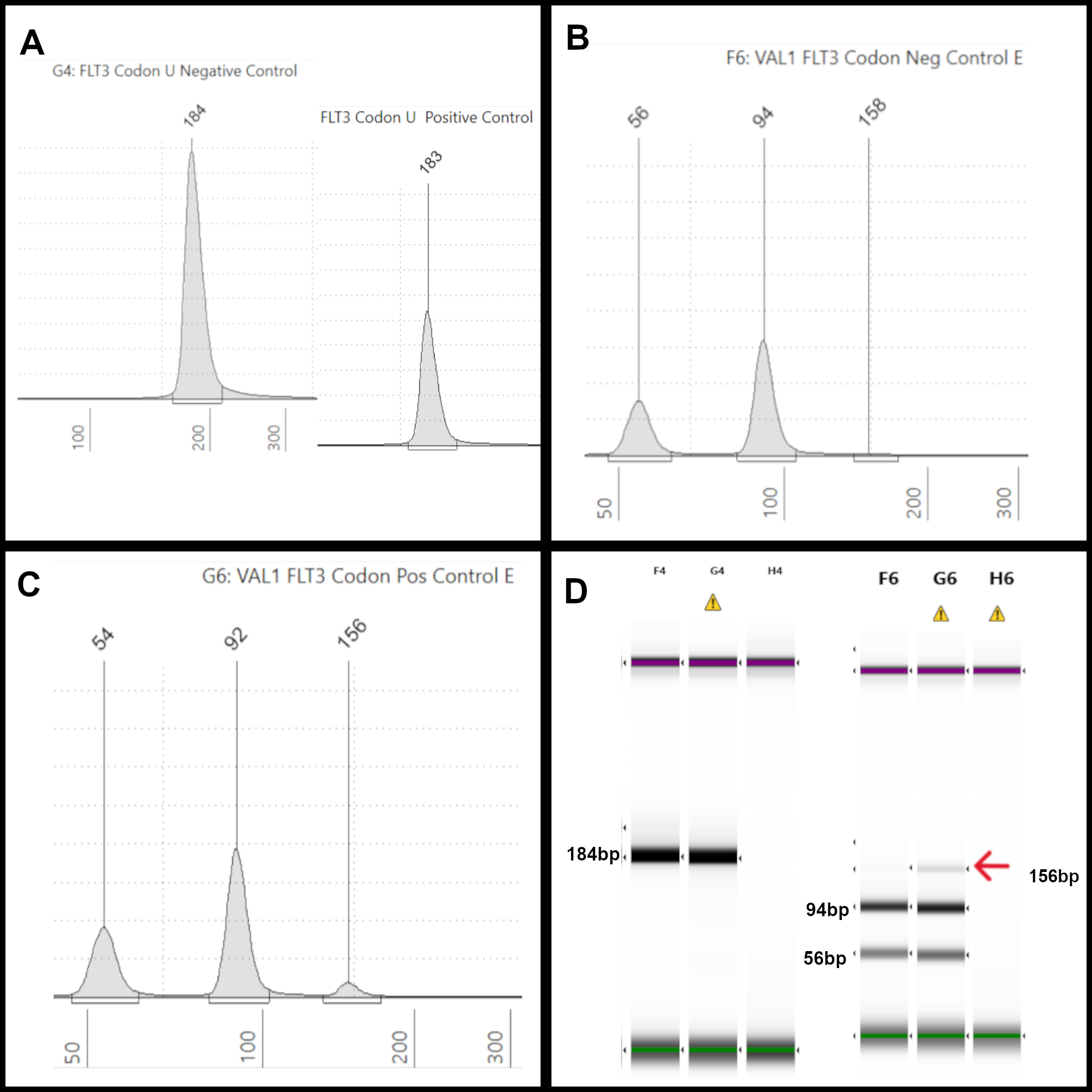

Supplement: Supplementary file 1 [file genes-16-00684-s001.zip › Supp fig 3 Codon controls.tif]

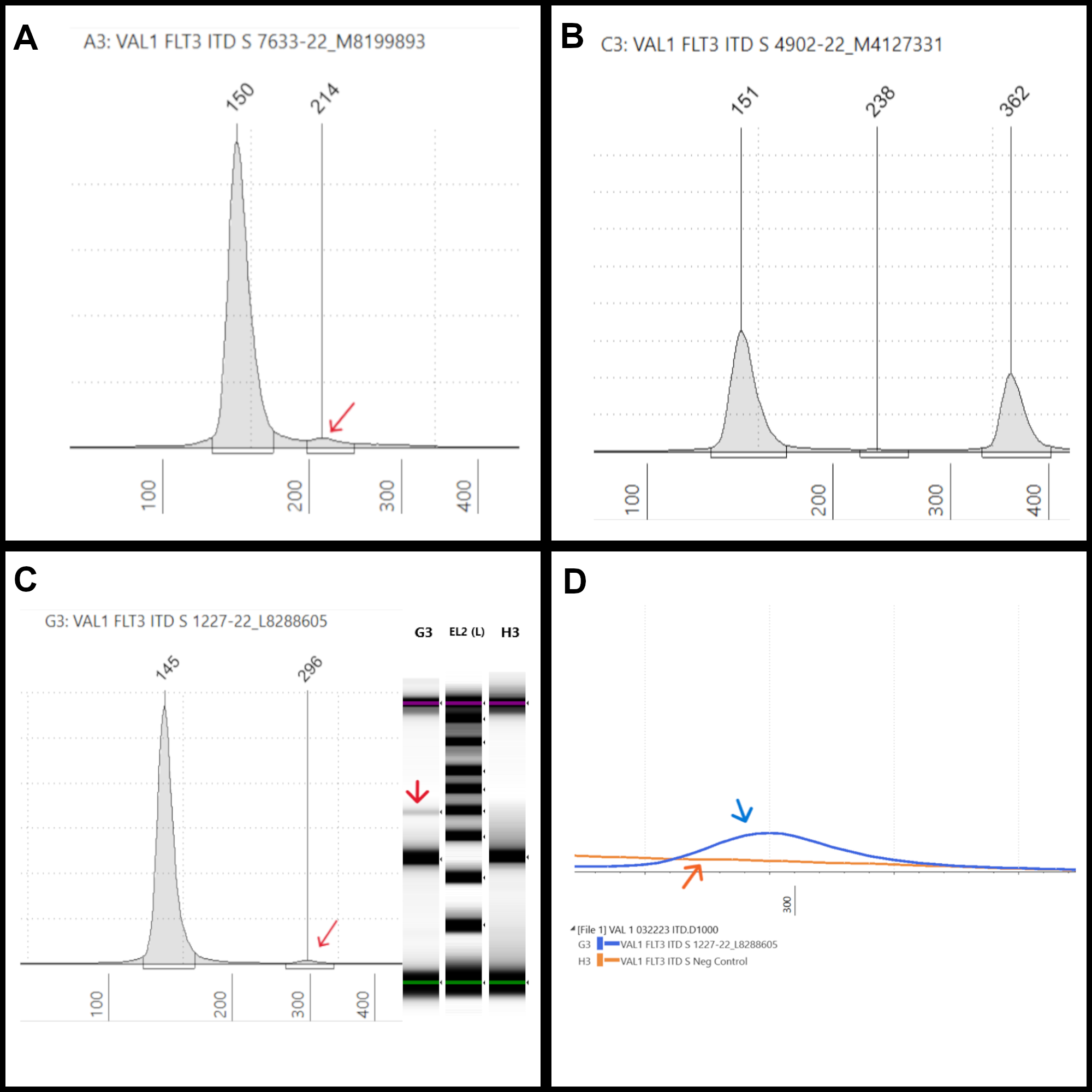

Supplement: Supplementary file 1 [file genes-16-00684-s001.zip › Supp fig 4 ITD cases.tif]

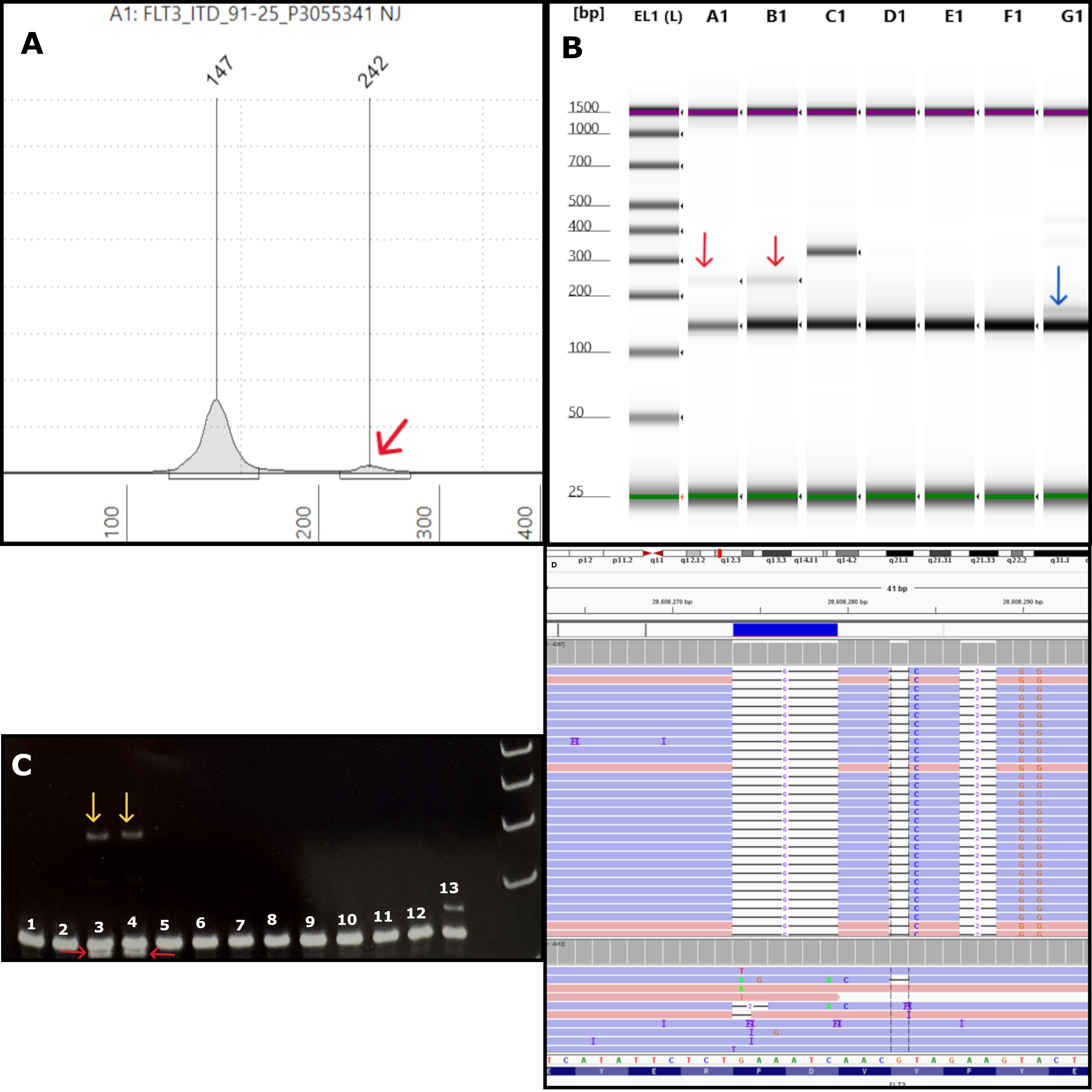

Supplement: Supplementary file 1 [file genes-16-00684-s001.zip › Supp fig 5 #91-25.tif]

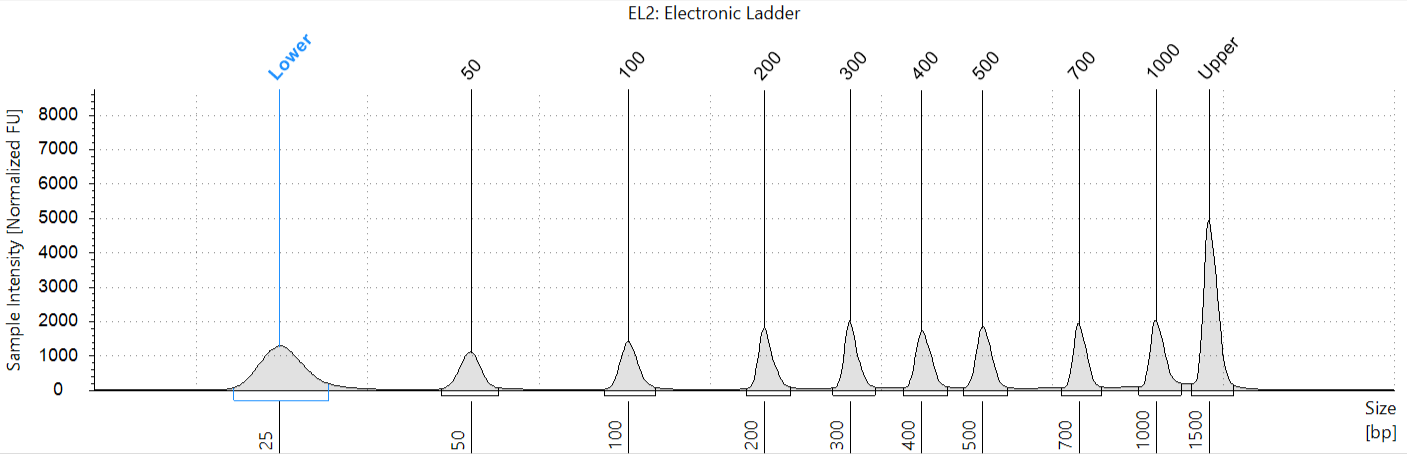

Supplement: Supplementary file 1 [file genes-16-00684-s001.zip › Supplemental Fig 1.tif]
